# Extraskeletal Myxoid Chondrosarcoma: A Comparative Study of Imaging and Pathology

**DOI:** 10.1155/2018/9684268

**Published:** 2018-06-07

**Authors:** Ling Zhang, Ruoning Wang, Rong Xu, Genggeng Qin, Lei Yang

**Affiliations:** ^1^Department of Radiology, Nanfang Hospital, Southern Medical University, Guangzhou 510515, China; ^2^Department of Radiology, Ningbo Sixth Hospital, Ningbo 315040, China; ^3^Department of Pathology, Nanfang Hospital, Southern Medical University, Guangzhou 510515, China

## Abstract

The purpose of this study was to achieve better understanding of extraskeletal myxoid chondrosarcoma (EMC). 13 cases of EMC confirmed by surgery biopsy were retrospectively studied. All patients underwent preoperative CT or/and MRI examinations. Among six patients who underwent preoperative CT examinations, six cases of lesions exhibited hypodensity on unenhanced image, three cases of tumor showed funicular spots or patchy calcification, and four cases of tumor did not show any obvious enhancement after enhanced CT scan. Among ten patients who underwent preoperative MRI examination, 8 cases of tumor revealed uniform or slight hyposignal intensity on T1WI, 10 cases of tumor demonstrated lobulated hypersignal intensity with multiple low signal intensity of interval septa on T2WI, and 5 cases of lesions indicated characteristic appearance: septa enhancement with tumor stroma between interval septa being unenhanced. EMC usually occurred at older men and at certain location such as limbs, trunk, and subcutaneous tissues. EMC usually exhibited low density mass (mostly 20-40HU) with calcification and in a portion of the cases showed light or no enhancement on CT. On MRI, EMC showed lobulated hypersignal intensity on T2WI with characteristic arc, septa, or interval septa enhancement.

## 1. Introduction

Extraskeletal myxoid chondrosarcoma (EMC) is a rare low-grade malignant mesenchymal neoplasm of uncertain differentiation characterized by abundant myxoid matrix located in the soft tissues. It affects mainly the soft tissues of the proximal end of long bones. Its incidence in the head and neck region is less than 5% [1, 2]. There is no evidence yet showing that EMC exhibits the feature of cartilaginous differentiation, although it is widely acknowledged that it could be pluripotent. It does not belong to one of the subtypes of chondrosarcoma and is a different disease from extraskeletal mesenchymal chondrosarcoma.

EMC demonstrate a strong tendency for local recurrence (37- 48%) and metastatic disease (50%), usually pulmonary [3]. However, the biological behavior of EMC is fairly indolent. Survival to discharge was more common among EMC patients than other soft tissue sarcoma patients [4]. EMC has a male preference, and this occurs in soft tissue area in patients who are more than 40 years old [5, 6].

Studies in the past were aimed at improving disease control in the surgical resection, radiotherapy, with some reported benefit from tyrosine kinase inhibitor sunitinib malate in the metastatic setting [7–10]. The literature contains few cases in which the imaging characteristics of EMC are discussed, and therefore a complete imaging and pathology interpretation of this disease is lacking.

In this article, we reported CT and MRI studies of 13 cases of EMC with corresponding pathological analyses. The purpose of this study was to achieve better understanding of EMC by describing its radiological and pathological characteristics. To our knowledge, this is the largest cohort of patients with EMC imaging characteristics that has been analyzed to date.

## 2. Materials and Methods

### 2.1. Patients Data

A total of 13 EMC cases were obtained from April 2005 to March 2017 at two hospitals, including our own. All cases were clinically diagnosed and went through surgical procedures, pathological analyses, and postsurgical follow-up in our hospital.

### 2.2. Imaging Acquisition

Computer tomography (CT) exams were performed in 7 cases before surgery. Dual energy CT (Siemens SOMATOM Definition) images were acquired with the following parameters: slice thickness 5-10 mm, tube voltage 120 kV, tube current 559 mA, pitch 3.2, gantry perpendicular to the CT table. Multiplanar reformatting of CT images was performed on a workstation (Advantage Workstation 4.3; GE Healthcare, Waukesha, WI, USA).

Magnetic resonance imaging (MRI) with and without contrast was performed in ten cases on a 3.0T MRI scanner (GE Signa Excite). Dedicated coils were used for imaging different body areas and the regions of interest. MR images were acquired using spin echo sequences—axial view: T1WI: TR/TE 440/8.2 ms, T2WI: TR/TE: 4000/142.5 ms, slice thickness: 5mm, NEX:4.0, FOV: 38cm×38cm, and matrix: 256×224~512×446. Fat-suppressed T1-weighted transverse images: the gadopentetate dimeglumine (Magnevist, Schering, Berlin, Germany) was intravenously injected. The dose was 0.1mmol/kg per patient and the injection rate was 2.0 ml/s—axial view: T1WI: TR/TE 560/8.0 ms, slice thickness: 6mm, FOV: 38cm×38cm, and matrix: 320×192~512×446; corona view: T1WI: TR/TE 560/8.2 ms, slice thickness: 5mm, FOV: 40cm×40cm, and matrix: 320×192~512×446.

### 2.3. Image Analysis

All images were analyzed by two board-certified radiologists, who specialize in musculoskeletal imaging. On CT images, tumor location, morphology, size, edge, density, and the presence of calcification of the tumor were evaluated. On MR images, tumor morphology, edge, signal intensity and enhancement, necrosis, hemorrhage, and peritumoral edema were evaluated. All imaging findings were correlated with pathological analyses.

### 2.4. Pathological Analysis

Tumor specimens obtained after surgical resection were fixed in 10% formaldehyde solution for 24 hours for dehydration, and the paraffin-embedded specimens were sliced and stained with hematoxylin and eosin (H&E). Immunohistochemistry: streptavidin-peroxidase-biotin (S-P) link staining with 3,3'-diaminobenzidine (DAB) color rendering was performed on some specimens. Cytoplasmic brown precipitate was considered to be positive. Histopathological characteristics were evaluated by a board-certified pathologist specializing in musculoskeletal specialty.

## 3. Results

### 3.1. Clinical Data

A total of 13 cases (6 males and 7 females) were collected, with ages ranging from 28 to 65 years with an average age of 41.9 years. The locations of EMC in each patient are listed in Table 1. Disease histories ranged from 1 week to 8 years, with the maximum tumor diameter measuring between 1.5 and 12 cm, with an average of 5.3 cm. Four patients with lesions located in the lumbosacral region of the spine, spinal canal, brachial canal, and nasopharynx had symptoms of nerve compression, nasal congestion, and nasal bleeding in early stages, whereas the other 9 patients did not report any pain or paresthesia, increase in skin temperature, or skin ulcers.

### 3.2. CT Findings

CT findings of all cases are listed in Table 1. Conventional CT scans were performed in 9 out of 13 cases (Figures 2, 4, and 6), of which 6 cases underwent enhanced CT scans. Streaky calcifications were seen in the center of 2 tumors (Figures 4(a) and 4(b)). In the 6 cases of enhanced CT scans, there was no obvious enhancement in the 3 lesions (cases 1, 10, and 13; Figures 2 and 6), with the CT number increasing to less than 10 HU, while one lesion (case 6) showed a mild enhancement with the CT number increasing to more than 10 Hu. The remaining two cases (cases 4 and 12) showed heterogeneous and moderate enhancements (Figure 4(b)), where the increase in CT number was higher than 20 HU, resulting in a clear distinction between the tumor and the surrounding tissues.

### 3.3. MRI Findings

**Figure 1:**
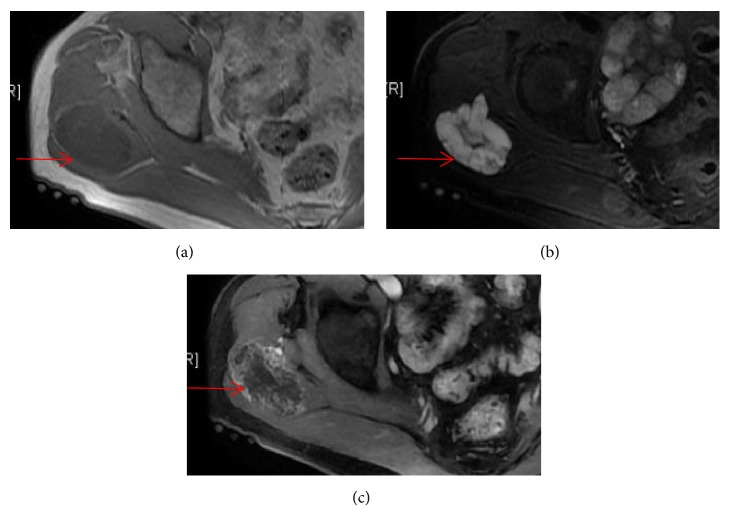
Images obtained from a 52-year-old man with EMC of the right gluteus. (a-b) T_1_WI and T_2_WI: the lesion (red arrow) demonstrated lobulated mass with hyposignal intensity on T1-weighted sequences and hypersignal intensity on T2-weighted sequences; it showed enhancing internal septation after injection of gadolinium (c).

**Figure 2:**
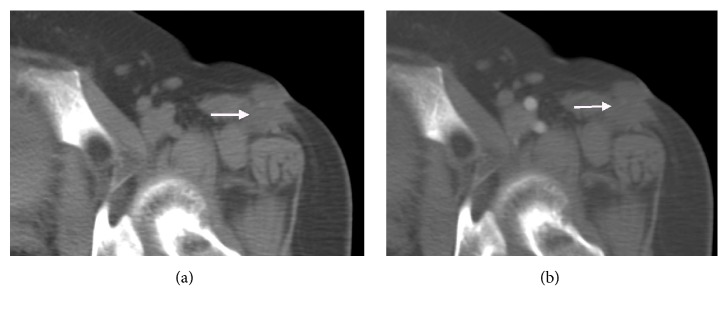
Images obtained from a 55-year-old woman with EMC of the left thigh. (a, b) Transverse CT scan showed low density, round mass without marked enhancement (white arrow).

**Figure 3:**
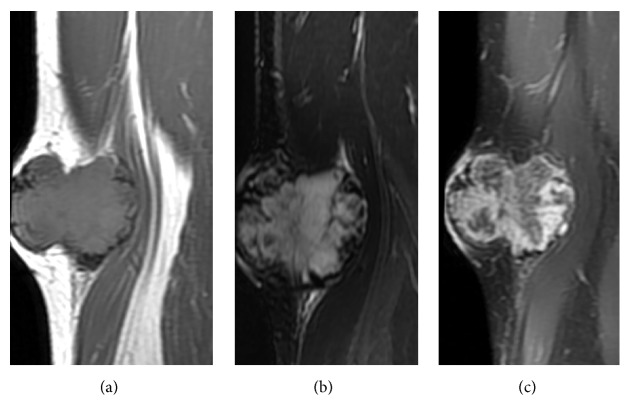
Typical radiological appearance from a 40-year-old man with EMC of the right thigh. An irregular-shaped soft tissue mass in right thigh with long T1 and T2 was observed (a, b). An enhanced MRI scan revealed an obvious uneven enhanced mass with radiated arrangement separation enhanced like spokes (c).

**Figure 4:**
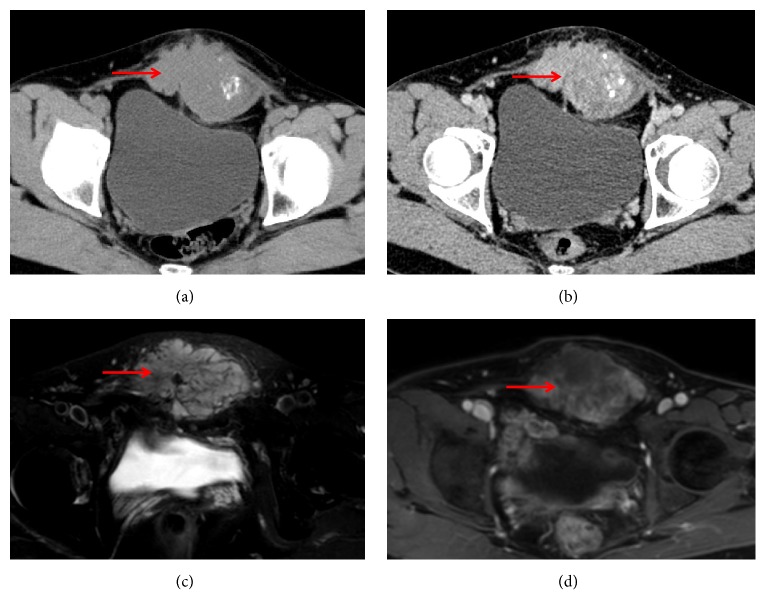
A 44-year-old woman with extraskeletal myxoid chondrosarcoma of the lower abdomen (red arrow). Transverse CT scan showed low density, irregular mass with spot-like calcification (a). Axial contrast-enhanced CT shows that this mass is with moderate enhancement (b). The lesion exhibited hypersignal intensity on T2-weighted sequences (c) and heterogeneous enhancement after injection of gadolinium (d).

**Figure 5:**
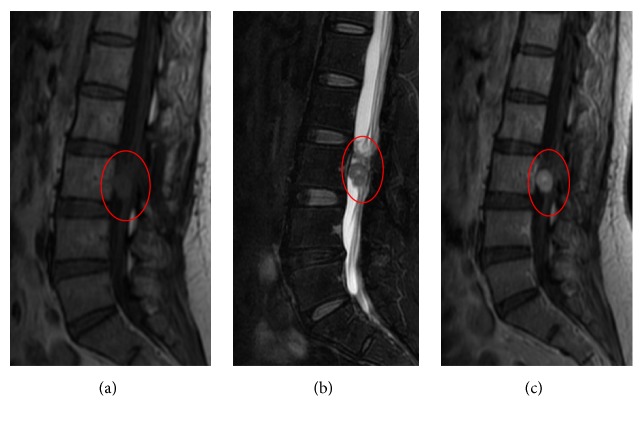
Images obtained from a 43-year-old woman with EMC of the spinal canal (red circle). MRI showed a mass in the spinal canal with both slight high signal on T_1_WI and T_2_WI (a, b). Gadolinium-enhanced T1-weighted fat-suppression images showed heterogeneous enhancement (c).

**Figure 6:**
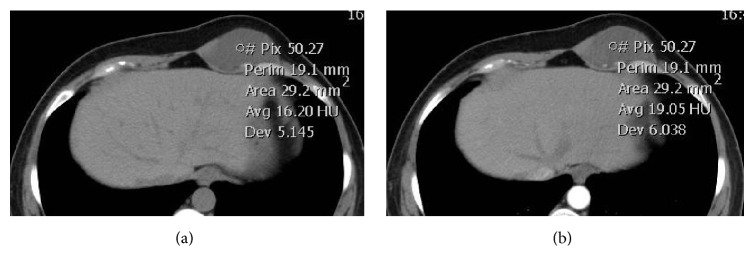
Typical radiological appearance from a 48-year-old man with EMC of the walls of the chest. Transverse CT scan showed low density, irregular mass ((a), CT value=16.2Hu) without marked enhancement ((b), CT value=19Hu in CT Contrast Enhancement).

**Figure 7:**
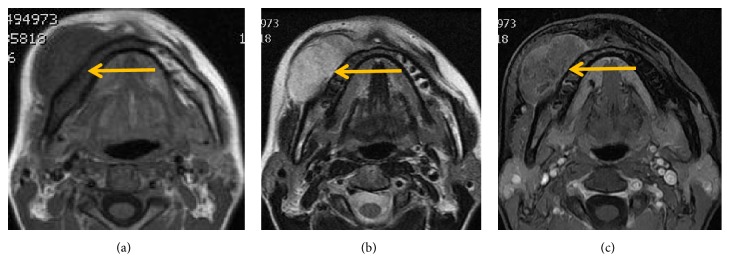
Imaging features (yellow arrow) from a 28-year-old woman of the right lower jaw. (a) T1-weighted MRI shows a uniform low signal mass. (b) T2-weighted MRI shows a heterogeneous high signal mass. (c) Gadolinium-enhanced T1-weighted fat-suppression images showed heterogeneous enhancement.

MRI findings of all cases are listed in Table 1. MRI exams were performed in 10 out of 13 cases (Figures 1, 3–5, and 7), of which 8 cases underwent contrast-enhanced MR scans. Eight cases (80%) appeared uniformly isointense or hypointense on T1WI (Figures 1(a), 3(a), and 5(a)), while the other 2 cases showed hypointense separate, multiple lobules (Figure 7(a)). On T2WI, tumors of the 8 above-mentioned cases showed bright signal intensity (Figures 1(b), 3(b), 4(c), 5(b), and 7(b)) and were separated into multiple septa with hypointensity. On contrast-enhanced MR images, signal enhancement was observed in the periphery of the septa, whereas the tumor stroma was not enhanced (Figures 1(c), 3(c), and 4(d)). In another case (10%), the peripheral tumor regions showed hypointensity while the tumor center showed hyperintensity on T1WI (Figure 5(a)). In addition, peripheral tumor regions were enhanced in the contrast-enhanced images. On T2WI of this case, the entire tumor regions appeared hyperintense with even brighter signal intensities in the tumor center (Figure 5(b)).

### 3.4. Pathological Findings

During surgical procedures, tumors showed a lobulated appearance with complete pseudocapsules. The gross specimen sections were composed of a plurality of jelly-like tumor nodules, separated by fibrous tissues between the nodules, partially exhibiting cystic degeneration, hemorrhage, and necrosis. Under optical microscope, numerous lobes were seen in the tumor, and the lobes were filled with mucinous stroma or cartilage myxoid stroma. Also a large number of tumor cells in the shape of a short spindle or circle that were distributed around the tumor periphery were observed. A small amount of tumor cells was embedded in the mucinous stroma, which was plentiful in the central region. In rare cases, tumor cells lost their self-adhesion and became distributed throughout the mucinous stroma.

### 3.5. Clinical Follow-Up

In 9 out of 13 cases, the patients had clinical follow-up within 10-33 months. Five of those patients have had no recurrence to date. Four of those patients had recurrent tumors in the original site within 5-19 months, and one patient had another recurrence after the second surgery. One patient died due to lung metastases.

## 4. Discussions

EMC was first described by Stout et al. in 1953 [11] in a discussion of various types of extraskeletal chondrosarcoma. Initially, it had various names such as extraskeletal chondrosarcoma and chordoma sarcoma. In 1972, Enzinger et al. [12] first proposed the concept of EMC. Brody [13] had thought that it was a unique low-grade malignancy with slow growth rate and a chronic history, different from typical chondrosarcoma both clinically and histopathologically. However, the parental lineage of EMC cells remains unclear. Therefore, EMC has been classified as a type of soft tissue tumor with uncertain differentiation according to the most recent edition of the World Health Organization Classification of Tumors of Soft Tissue and Bone [14].

Recent statistics show that EMC cases have a high rate of local recurrence, metastasis, and patient mortality rate [15] and is therefore classified as an intermediate grade malignancy. There are rare instances when spontaneous regression of metastatic lung tumors of EMC occurs without any treatment [16]. EMC is rare, accounting for less than 3% of soft tissue tumors. It mainly occurs in adults, with an average age of about 54 years (ages range from 29 to 73 years). The occurrence ratio between male and female is 2:1 [17]. Two-thirds of EMC tumors are found to occur in the limbs, especially in the thigh and popliteal fossa, with an average diameter of about 9.3 cm (3.3-18cm).

Our data shows that the average age of onset of the EMC patients was about 41.9 years. In six cases (2/3) EMC was located in the hips, thighs, knees, and wrists, similar to those reported in the literature. Atypical EMC locations included the nasopharynx, lower jaw, chest, abdomen, and spine. The average diameter of EMC was found to be 5.5 cm (1.5~8.9cm). Depending on their locations, EMC tumors were more likely to be diagnosed when patients suffered from nerve compression symptoms. These tumors exhibited smaller diameters. EMC has a 5-year survival rate of 100% and a 10-year survival rate of approximately 70% [18, 19]. It has a recurrence rate and metastasis rate of 48% and 46%, respectively. The prognosis may not be as good as previously reported. Four cases in the study group had local recurrences within 2-4 years after surgery.

There was no specificity of CT findings for primitive EMC [17, 19–21], mostly shown as heterogeneous tumors in soft tissues. The CT numbers of the tumors, mostly within 20-40 HU, were generally lower than the surrounding normal muscular tissues. Typically, distinctive enhancements were not observed in enhanced CT scans. Neena [10] mentioned that EMC showed mild enhancement in enhanced CT scans. Zhang [22] reported that EMC revealed an irregular-shaped soft tissue mass with clear boundary and uniform density. The adjacent bone of the mass did not show obvious absorption and destruction. In this study, the densities of the tumors were mostly lower than those of the adjacent muscles, and the densities of the center of the tumors were even lower, which is consistent with the literature [8]. These findings were mainly related to the tumor's histological constitution. Microscopically, a large number of tumor cells took the shape of silk ribbons and spoked wheels in the tumor periphery, while the central region was rich in mucinous stroma, with few tumor cells and little angiogenesis. Because mucinous stroma accounted for most of the tumor mass, the CT images showed lower density in the central region than in the periphery. Also, little enhancement was achieved in enhanced scans because of the low amount of angiogenesis in the central region. Little or no tumor enhancement was observed in this study. If EMC recurs repeatedly, the tumor regions with solid stroma and little mucus will increase [21], resulting in more tumor cells, more obvious atypia, and more profuse angiogenesis within the tumor. In such cases, more enhancement for the tumor will be observed during enhanced CT scans. These tissue changes led to significant enhancement in two cases of recurrence in this study group. Moreover, CT scan is more sensitive than MRI for detection of calcifications. CT scan may demonstrate an ill-defined cloud-like matrix with calcified whorls and arcs [23, 24].

Tumor calcification, additionally, had not been reported in the literature. However, different forms of calcification were found within the tumors in 3 cases in this study, one of which was a large tumor located in the right thigh and the pelvic areas. Calcification was mainly shown as a large amount of spots and patches in the tumor peripheries. Under microscope, tumor stromal necrosis was observed, leading to a secondary calcium deposition. No calcium was present in the central area with mucus.

EMC was characterized by the presence of cartilage matrix on MR images [22, 25, 26]. The mass showed a lobulated appearance with iso/hypointensity to muscle on T1WI, hyperintensity on T2WI, and it was usually separated into multiple lobules by multiple septa appearing hypointense. The signal intensity was higher in the myxoid areas than in the solid areas, and the calcified areas appeared hypointense on T2WI. In contrast-enhanced images, peripheral, septal, or heterogeneous enhancement patterns were observed. Seven of 10 cases in this study demonstrated the typical MR features described above, and these masses had large sizes in the subcutaneous tissue of the extremities and trunk. Only 3 cases did not present those typical characteristic MR features. In those 3 cases, masses had unusual sites of origin, such as the vertebral canal, carpal tunnel, and the right lower jaw. The appearance of lobulated nodules was not seen on MRI when the myxoid stroma was not arranged in a nodal architecture.

It is essential to differentiate EMC from extraskeletal mesenchymal chondrosarcoma, which is also rare. As the histologic overlap between EMC and other epithelioid and myxoid soft tissue neoplasms is noteworthy, especially at the high-grade end of the spectrum, it is difficult to exclude the fact that some of the tumors included in these series may represent alternative diagnoses [27, 28]. Extraskeletal mesenchymal chondrosarcoma mainly grows in the muscle, with some reported occurrences in the heart, nasal cavity, mediastinum, and kidney. Pathological analyses reveal that extraskeletal mesenchymal chondrosarcoma comprises undifferentiated cartilage islands. The cartilage islands contain the primitive vascular structures, shown as punctate calcification clumps on CT. They have abundant blood supply and therefore significant enhancements on enhanced CT scans. Compared with EMC, extraskeletal mesenchymal chondrosarcoma shows typical nodular hyperintensity, in conjunction with patchy enhancement patterns, but barely do they show nodular ring enhancement patterns on enhanced CT scans. It is also important to differentiate EMC from myxoid liposarcoma and soft tissue myxoma. Myxoid liposarcoma shows clear tumor boundaries, shown on T2WI as multiple lobulated tumors with hyperintensity, but lacking the wheel and spoke-like hypointense septa. Myxoid liposarcoma generally has homogeneous or inhomogeneous enhancements in enhanced scans, while EMC mostly has boundary enhancements and septa enhancements. Soft tissue myxoma appears hypointense on T1WI. The ring of fat mass and edema around the tumor are highly suggestive of this disease [25] on the water-fat separation MR images. In addition, neither of these two diseases has calcification.

One limitation of this study is that it is a retrospective and multicenter study due to the rareness of EMC, and a single inspection method is not sufficiently conducive to the determination of EMC. The second limitation is that only 4 cases had both CT and MRI scans, with one case lacking an enhanced CT scan and another case lacking an enhanced MRI scan. Two cases (cases 2 and 13) did not show definite enhancements on CT scans (CT number increases were less than 10 HU). It is unknown whether enhancement and enhancement patterns on MRI would have been observed due to the lack of MRI scans. Future large-scale studies need to be performed to further improve the characterization of EMC.

## 5. Conclusion

Preliminary conclusions can be reached by combining the literature and the findings in this study. EMC is likely to exist at certain ages in certain genders (older men) and in specific body regions (limbs, trunk, and subcutaneous tissues). The existence of EMC is also characterized by tumors with low density (mostly 20-40 HU) and calcifications on CT images, mild or no enhancement on enhanced CT images, lobulated hyperintensity on T2WI, and peripheral or septal enhancements on contrast-enhanced MRI. However, EMC cannot be distinguished from common extraskeletal chondrosarcoma using MRI only. Therefore, diagnosing this disease with both CT and MRI exams is recommended.

## Figures and Tables

**Table 1:** The clinical and imaging manifestations of EMC.

No	Age	Sex	location	Medical history	size (cm)	Manifestation of CT	CT value (Hu)	CT enhancement	T1WI	T2WI	Internal mass enhancement_a_
1	52	M	Right gluteal	20+ days	7.5x7	/	/	/	Slight low signal	Strongly high signal, lobulated	Enhancing internal septation
2	55	F	Left thigh	1 year	1.6x2.2	Low density, round mass	15-25	Minimal (<10Hu)	/	/	/
3	40	M	Right thigh	6 years	7.5x7.1	/	/	/	Iso signal, with dark internal septation	Strongly high signal, lobulated	Enhancing internal septation
4	44	F	lower abdomen	1 year	8.9x8.5	Isodensity, lobular mass, with calcification	37-40	Moderate enhancement, heterogeneous	Iso signal, with dark internal septation	Strongly high signal, lobulated	Enhancing internal septation
5	43	F	Spinal canal	3 years after operation	1.1x1.5	/	/	/	Slight high signal	Iso signal	Heterogeneous
6	54	M	Right pars nasalis pharyngis	20 days	4.0x2.7	Isodensity, irregular mass	24-38	Minimal (<10Hu)	/	/	/
7	65	M	Right carpal canal	8 years	1.5x1.8	/	/	/	Iso signal	high signal, with dark internal septation	/
8	47	M	Left buttocks	5 years	8x6	/	/	/	Iso signal, with dark internal septation	Strongly high signal, lobulated	Enhancing internal septation
9	45	F	Right knee	1 week	8.0x6.6	Low density, irregular mass	23-28	/	Iso signal, with dark internal septation	Strongly high signal, lobulated	Enhancing internal septation
10	48	F	Walls of the chest	3 years	3.2x4.1	Low density, irregular mass	16-20	Minimal (<10Hu)	/	/	/
11	28	F	Right lower jaw	1 month	4.6x3.7	/	/	/	Slight low signal	High signal	Heterogeneous
12	43	M	Right lower limb, pelvis	3 years	12x10	Uneven density, irregular mass, with calcification	/	Moderate (>20Hu)	Mixed signal	Strongly high signal, lobulated	Rim enhancement
13	33	F	Right lumbosacral portion	2 months	4.5x2.5	Uneven density, irregular mass	/	Minimal (<10Hu)	Slight low signal	Strongly high signal, lobulated	/
